# Potential Metabolic Drug–Drug Interaction of *Citrus aurantium* L. (*Rutaceae*) Evaluating by Its Effect on 3 CYP450

**DOI:** 10.3389/fphar.2018.00895

**Published:** 2018-08-31

**Authors:** Lu Zhou, Man Cui, Linlin Zhao, Dongsheng Wang, Tao Tang, Wenbo Wang, Sheng Wang, Huiyong Huang, Xinjian Qiu

**Affiliations:** ^1^Department of Integrated Traditional Chinese and Western Medicine, Xiangya Hospital, Central South University, Changsha, China; ^2^Medicine Service Section, The Second Affiliated Hospital of Shandong University of Traditional Chinese Medicine, Shandong, China; ^3^Health Management Center, Third Xiangya Hospital of Central South University, Changsha, China; ^4^Provincial Key Laboratory of Traditional Chinese Medicine Diagnostics, Hunan University of Chinese Medicine, Changsha, China

**Keywords:** *Fructus aurantii*, CYP1A2, CYP3A4, CYP2E1, drug–drug interaction

## Abstract

**Aim:** *Fructus aurantii* (FA) is widely used in clinic as an expectorant and digestant herb in traditional Chinese medicine and proven to have a variety of pharmacological functions. FA is close to grapefruit either by botanical taxonomy or by their same components (flavonoids, etc.) and grapefruit has been proven to cause drug–drug interaction when co-administrated with CYP3A4 substrates. Besides, FA contains many compounds, such as flavonoids, which have been reported to impact the expressions of CYP450. However, the effect of FA on CYP450, whose change may affect drug safety and clinical efficacy attributed to drug–drug interaction, still remains unknown.

**Methods:** The protein, mRNA expression and enzyme activity of CYP1A2, CYP3A4, and CYP2E1 in rat were determined by Western Blotting, RT-PCR method, the cocktail method, respectively, after orally administration of FA in succession for 7 days. CYP1A2, CYP3A4, and CYP2E1 mRNA expression were investigated in HepG2 cells following FA-medicated serum incubation for 24 h.

**Results:** In rat, compared to the control group, CYP1A2, CYP3A4 protein, and mRNA expression were significantly induced consistent with the corresponding CYP activities; the protein expression of CYP2E1 was significantly upregulated, while the corresponding mRNA expression and enzyme activity showed no significant change. In HepG2 cells, compared to the control group, the mRNA expression of CYP1A2 and CYP3A4 was up-regulated statistically while CYP2E1 mRNA expression was not significantly induced or inhibited.

**Conclusion:** FA may be a potential slight inducer of CYP1A2 and CYP3A4 and is unlikely to impact CYP2E1 until clinical researches are conducted.

## Introduction

*Fructus aurantii* (FA, Zhiqiao in Chinese), the dried and immature fruit of *Citrus aurantium* L. (*Rutaceae*) or its cultivars gathered from July, is used as an expectorant and digestant herb in traditional Chinese medicine, which is cited in Chinese Pharmacopoeia (1 part) of 2015 version. It is widely used in clinic as one component of several formulae, such as Xue-Fu-Zhu-Yu Tang (Liu et al., 2004), decoction of *Bulpleurum falcatum* L. and FA (Li et al., 2004), the formulae of FA and Magnolia bark which has produced relatively favorable effect to functional dyspepsia (Huang et al., 2011; Xiong et al., 2015), Chaihu-Shugan-San (Fan et al., 2012), and Wei-Chang-An-Wan (Liu et al., 2013). FA extract is reported to have a wide range of potential therapeutic effects, such as hypocholesterolemic action (Kim et al., 2011), anti-tumor action (Zhou et al., 2007), prokinetic action (Huang et al., 2011; Zhang et al., 2012), anti-ischemic action (Kang et al., 2007), and anti-depressive action (Fan et al., 2012; Zhang et al., 2012). It has been reported that FA reduces portal pressure in portal hypertensive rats (Huang et al., 1995) and has been frequently used to treat cardiovascular symptoms in Asian countries (Kang et al., 2007; Kim et al., 2011).

The Cytochrome P450 enzymes (CYP450), monooxygenases metabolizing xenobiotics and endogenous substrates, are involved in approximately 80% of oxidative drug metabolism and account for almost 50% of the overall elimination of commonly used drugs (Wilkinson, 2005). Among the Cytochrome P450 enzymes, families 1–3 constitute almost half of total CYPs in mammals (Bièche et al., 2007; Tydén et al., 2014), and CYP1A2, CYP2E1, and CYP3A4 were mainly expressed in the liver (Bièche et al., 2007), which is the major site of Cytochrome P450 mediated metabolism (Wilkinson, 2005). CYP450 was reported to mediate drug–drug interaction. The well-known example is that grapefruit juice increases the bioavailability of various drugs due to the inhibition of CYP3A4 metabolism (Yoshida et al., 2005).

The FA is confirmed to contain flavonoids, furanocoumarins, essential oil, alkaloids, coumarins, and microelement. These compounds are reported to interact with CYPs in many literatures. It is known that flavonoids, considered to be the major bioactive constituents of FA (Tong et al., 2012), can either inhibit or activate human Cytochrome P450 depending upon their structures, concentrations, and experimental conditions (Hodek et al., 2002). Furanocoumarins in Citrus crude drugs is found to inhibit not only CYP3A activity but other CYP subtypes, which may cause *in vivo* drug interactions (Guo et al., 2001). Thus, we wonder whether FA is potential to exert influence on CYPs and cause drug–drug interaction for reasons as follow: on one hand, FA contains the compounds which were demonstrated to effect CYPs expressions; on the other hand, FA is close to grapefruit either by botanical taxonomy or by their same components (flavonoids, furanocoumarins, etc.) and grapefruit has been proven to influence CYP3A4 contributing to drug–drug interaction.

However, reports on the FA related to influence on CYPs were rarely found. Considering the extensive clinical application of FA and the poor information of the impact of FA on CYPs, it is beneficial that a study, whether FA has effects on CYPs, can be carried out. In this paper, hepatic CYP1A2, CYP2E1, CYP3A4 protein expression, mRNA expression, and enzyme activity were detected in rat after oral administration of FA extract by Western blot, RT-PCR, and cocktail method, respectively. The cocktail method adopted probes including caffeine (CYP1A2), dapsone (CYP3A4) and chlorzoxazone (CYP2E1). Their mRNA expression in HepG2 cells was analyzed by RT-PCR based on HepG2 cell system and serum pharmacological method. For the sake of safety profiles and drug efficacy, this study is worth conducting.

## Materials and Methods

### Materials

Dulbecco’s modified Eagle’s medium (DMEM), fetal bovine serum and 0.25% Trypsin-EDTA were purchased from Gibco Life Technologies^TM^. The 3-(4, 5-dimethyl-2-thiazolyl)-2, 5-diphenyl-2H-tetrazolium bromide (MTT) were obtained from Sigma–Aldrich Co. Anti-rabbit horseradish peroxidase-conjugated secondary antibody, anti-mouse horseradish peroxidase-conjugated secondary antibody, anti-β-actin antibody, anti-Cytochrome P2E1 antibody, and anti-CYP3A4 antibody were obtained from Abcam. Anti-CYP1A2 rabbit polyclonal antibody was purchased from Sangon Biotech (Shanghai) Co., Ltd. Pierce^TM^ BCA Protein Assay Kit and RevertAid First Strand cDNA Synthesis Kit were purchased from Thermo Fisher Scientific. All-in-One^TM^ qPCR Mix Kit was purchased from GeneCopoeia. TRIzoL Reagent was bought from Invitrogen. Western blotting luminal reagent was obtained from Santa Cruz Biotechnology, Inc. Acetonitrile, methanol, formic acid, and reference substances (caffeine, chlorzoxazone, dapsone, naringenin, hesperetin, and meranzin hydrate) were HPLC grade, other reagents were analytical grade.

### Plant Material and Preparation of *Fructus aurantii* Extract

The herb FA was purchased from the pharmacy of Xiangya Hospital of Central South University, Changsha, China, and authenticated. The voucher specimen (No. 2014070802, Jiangxi, China) was deposited in the Laboratory of Ethnopharmacology, Xiangya Hospital of Central South University. The procedures of extraction were simply described as follow: Herb FA material was thoroughly decocted twice in water (1:8 and 1:6, w/v, respectively) for 30 min at 100°C after soaking for 30 min. The decoction in both times were collected, blended, and filtered through eight layers of gauze twice before concentration with a vacuum rotary evaporator at 56°C. The concentrations of the water extracts we obtain were 0.625, 1.25, and 1.875 of raw material per ml, respectively. The final FA extracts were stored at -20°C prior to use.

### Animals

Male Sprague–Dawley rats, weighing 200 ± 20 g, were supplied by Animal Experimental Center in Kaifu District (Changsha, China). This study was strictly implemented according to the Regulations for the Administration of Affairs Concerning Experimental Animals (1988), which were approved by the Animal Experimental Center for Central South University (Changsha, China). The rats were caged (3 rats per standard cage) with free access to food and tap water under strictly controlled conditions (room temperature: 25 ± 2°C, relative humidity: 50 ± 10%, 12 h light/dark cycle). The animals were allowed to adapt to the new environment for 3 days before the commencement of this experiment.

### Effect of FA on the CYPs Protein and mRNA Expression in Rats

#### Treatment With Animals and Preparation of Liver Tissues and FA-Containing Serum Samples

The rats were randomly distributed into four subgroups (six each) and then orally administrated saline or FA extraction for 7 days in succession (once per day): the control group treated with saline, low-dose group received 10 g kg^-1^day^-1^ FA extraction, medium-dose group received 20 g kg^-1^day^-1^ FA extraction and high-dose group received 30 g kg^-1^day^-1^ FA extraction. Rats were anesthetized with chloral hydrate (3–4 ml kg^-1^ bw) and then sacrificed 1 h later after the last administration. The blood samples were collected into non-heparinized tubes by cardiac puncture. Followed centrifugation at 2500 rpm min^-1^ for 20 min at 4°C, which the serums were separated, the blood samples were kept at room temperature for about 2 h. The serums were decanted and then filled with 0.22 μm cellulose acetate membrane twice. After that, the serums were calefied by 56°C water for 30 min and then stored at -20°C until use. Liver sample from each animal was excised, perfused with ice-cold 0.9% (w/v) sodium chloride to remove blood, weighted, and stored at -80°C.

#### Western Blot Analysis

The liver samples were lysed with RIPA/PMSF (100:1, v/v) lysis buffer for 30 min on ice, following being grounded into powder with the help of liquid nitrogen. Total protein was separated by centrifugation at 12000 rpm min^-1^ at 4°C for 30 min. The protein concentrations were measured by using the BCA protein assay kit according to the manufacturer’s protocol. Protein fully mixed with loading buffer (4:1, v/v) and then denatured at 100°C for 5 min. Equal amounts of protein (64 μg lane^-1^) were separated by SDS–PAGE gel with 5% (v/v) stacking gel and 10% (v/v) resolving gel. Proteins were transferred onto PVDF membranes and blocked with 5% BSA in Tris-buffered saline with 0.1% Tween-20 (TBST) for 2 h at room temperature. Subsequently, membranes were incubated overnight at 4°C with 1:1000, 1:200, 1:4000, and 1:5000 dilutions of monoclonal antibodies for CYP2E1, CYP1A2, CYP3A4, and β-actin, respectively. Membranes were washed thrice with TBST and then incubated for 1 h at room temperature with 1:5000 dilutions of anti-rabbit horseradish peroxidase-conjugated secondary antibodies for CYP2E1, CYP1A2, and CYP3A4 and with a 1:5000 dilution of anti-mouse horseradish peroxidase-conjugated secondary antibody for β-actin. Again, membranes were rinsed three times. Finally, proteins were detected with an enhanced chemiluminescence (ECL) kit, followed imaging through ChemiDoc^TM^ XRS Imaging System (Bio-Rad Laboratories, United States). The protein bands were scanned by Image Lab^TM^ software (version 4.0, Bio-Rad Laboratories, United States). Quantity One^®^ software was used for the densitometric analysis of the bands. All isoforms were normalized to β-actin and data were presented as the relative protein expression.

#### Real-Time PCR Analysis

Total RNAs were extracted from the rat liver samples with Trizol Reagent in accordance with the manufacturer’s instruction. The concentration of RNA was quantified and the quality of RNA was assessed through the ratio of the absorbance at 260 and 280 nm. Subsequently, 2 μg of RNA was reversely transcripted to cDNA using RevertAid First Strand cDNA Synthesis kit, and then the cDNA was amplified using All-in-One^TM^ qPCR Mix Kit. The sequences of the primers are shown in **Table 1**. The reactions were conducted with a total reaction volume of 20 μl containing 2 μl of template diluted (1:10). The program of RT-PCR was set as follow: 95°C for 10 min, 40 cycles of 95°C for 15 s, 57°C for 20 s, and 72°C for 45 s. The relative mRNA expression was analyzed by the 2^-ΔΔ^*^C^*^t^ method.

**Table 1:** The RT-PCR oligonucleotide primers in rat.

Gene	Primer	Sequence (5′–3′)	PCR product (bp)
CYP2E1	Forward	CCTACATGGATGCTGTGGTG	171
	Reverse	CTGGAAACTCATGGCTGTCA	
CYP3A4	Forward	TCTGTGCAGAAGCATCGAGTG	253
	Reverse	TGGGAGGTGCCTTATTGGG	
CYP1A2	Forward	TGTCACCTCAGGGAATGCT	212
	Reverse	GACCACCGTTGTCTTTGTAG	
β-actin	Forward	TCGTGCGTGACATTAAAGAG	134
	Reverse	ATTGCCGATAGTGATGACCT	

### Effect of FA on the CYPs Activities in Rats

The rats were randomly divided into two subgroups (six each) and then orally administrated saline or FA extraction for 7 days in succession (once per day): the control group treated with saline and experimental group received 30 g kg^-1^day^-1^ FA extraction. On the 8th day morning, 5 ml kg^-1^ of probe cocktail solution, containing caffeine (10 mg kg^-1^), dapsone (10 mg kg^-1^), and chlorzoxazone (20 mg kg^-1^) suspended in CMC-Na solution, was then given to the rats in each group by intragastric administration. Blood samples were collected into heparinized tubes from the rat tail vein at a range of time-points (0, 5, 15, 30, 45, 60, 90, 120, 180, 240, 360, 480, 720, and 1440 min) after oral administration of three probe drugs, followed immediate centrifugation at 3500 rpm min^-1^ for 15 min to obtain 100 μl rat plasma samples. These were stored at -20°C until analysis.

To each 100 μl of rat plasma sample, the internal standard (30 μl 7.6 μg ml^-1^ sulfamethoxazole) was added before extraction with 370 μl acetonitrile, vortexing for 1 min and centrifugation at 5000 rpm min^-1^ for 5 min. A total of 400 μl supernatant was evaporated to dryness under a stream of nitrogen. The residue was dissolved in 50% methanol followed centrifugation at 12000 rpm min^-1^ for 5 min. A total of 10 μl of the solution was injected into UPLC analysis.

The standards of these components (caffeine, dapsone, and chlorzoxazone) were obtained from the National Institute for Food and Drug Control. A mixed stock standard solutions were added to blank plasma to yield seven different concentrations: for caffeine, they were 16.667, 8.333, 4.167, 2.083, 1.042, 0.521, and 0.260 μg ml^-1^; for dapsone, they were 3.300, 1.650, 0.825, 0.413, 0.206, 0.103, and 0.052 μg ml^-1^; for chlorzoxazone, they were 22.000, 11.000, 5.500, 2.750, 1.375, 0.688, and 0.344 μg ml^-1^, and for sulfamethoxazole, they were 119.000, 59.500, 29.750, 14.875, 7.438, 3.719, and 1.859 μg ml^-1^. The samples were extracted and analyzed according to the sample preparation procedures described above.

Chromatographic analysis was carried out on a Waters Acquity UPLC system, which equipped with a quaternary solvent delivery system, an auto-sampler fitted with a 10 μl loop and a PDA optical detector for ultraviolet wavelengths (190–480 nm). The separation was performed on Waters BEH (R) C18 (50 mm × 2.1 mm, 1.7 μm). The mobile phase consisted of acetonitrile (A) and 0.5% acetic acid in water (B). The elution gradient was as follows: 0–2 min, 20–30%A; 2–3 min, 30–40%A; 3–4 min, 40–40%A; 4–5 min, 40–50%A; 5–7 min, and 50–20%A. The flow rate was 0.2 ml min^-1^. The column temperature was maintained at 40°C. UV absorbance wavelengths were detected at 237, 293, and 281 nm for caffeine, dapsone, and chlorzoxazone, respectively.

### Effect of FA-Medicated Serum on CYPs mRNA Expression in HepG2 Cells

#### Serum Sample Preparation for UPLC Analysis

Naringenin (catalog No. 130330), hesperetin (catalog No. 130816), and merazin hydrate (catalog No. 20090118) in the FA-medicated serum were monitored by UPLC-PDA method. The standards of these components that were purchased from Chengdu must be Bio-Technology Co., Ltd. Stock solutions were separately prepared by dissolving the accurately weighed these standard reference compounds with methanol. A mixed stock solution was obtained by mixing the three stock solution above, and giving a final concentration of 3.555 μg ml^-1^ for naringenin, 0.236 μg ml^-1^ for hesperetin, and 3.300 μg ml^-1^ for meranzin hydrate, respectively. All the solutions were stored at 4°C refrigerator until use.

An aliquot (200 μl) of low-dose group rat serum was precipitated protein by vortexing vigorously with acetonitrile (500 μl) for 2 min. The supernatant was decanted and then evaporated to dryness under a stream of nitrogen after centrifugation at 10000 *g* for 15 min at 4°C. The residue was dissolved in methanol (100 μl), vortexing for 3 min, and then filtered through a 0.2-μm film. Following being ultrasonated for 1 min and repeating the same centrifugation procedure, an aliquot (10 μl) was injected onto the UPLC column for analysis.

Chromatographic analysis was conducted on the Waters Acquity UPLC system. The mobile phase consisted of acetonitrile (A) and 0.5% acetic acid in water (B). The gradient program was as follows: 0–2 min, 20–20% A; 2–4 min, 20–30% A; 4–5 min, 30–30% A; 5–7 min, 30–40%; 7–8 min, 40–40% A; 8–10 min, 40–20% A. The flow rate was 0.2 ml min^-1^, and the column temperature was set at 40°C. UV absorbance was detected at 284 nm using a photodiode array detector.

#### Cell Culture

HepG2 cells were cultured in the humidified incubator with a 5% CO_2_ atmosphere at 37°C. The medium used for cell culture, which was changed regularly, consisted of 89% DMEM, 0.5% penicillin (100 units ml^-1^), 0.5% streptomycin (100 g ml^-1^), and 10% fetal bovine serum. Cells were used for experiments when the confluence reached to 90%.

#### MTT Assay

The cytotoxic effects of FA in HepG2 cells were assessed by the MTT assay. Briefly, HepG2 cells were seeded at a density of 10,000 cells per well in 96-well cell culture plates. After 24 h treatment, the cell culture mediums were discarded and replaced with the new medium containing 10% rat serum (saline-treated rat serum, FA-treated rat serums) instead of 10% FBS. Culture medium without HepG2 cells was considered as the blank group and culture medium containing saline-treated rat serum with HepG2 cells as control group. After 24 h treatment in the cell incubator, MTT (5 mg ml^-1^, diluted with PBS buffer) were added into each well. Following another 4 h of incubation, the supernatants were removed and DMSO was added. After the plate was shaken for 10 min, the absorbance was recorded at the wavelength of 570 nm by an enzyme-labeling instrument:

$$\begin{array}{c} \mathrm{Cell}\;\mathrm{viability}\;\left(\%\right)=\left({\mathrm{OD}}_{FA-treated\;\mathrm{rat}\;\mathrm{serum}\;\mathrm{group}}-\;{\mathrm{OD}}_{\mathrm{blank}\;\mathrm{group}}\right)/\left({\mathrm{OD}}_{\mathrm{control}\;\mathrm{group}}-\;{\mathrm{OD}}_{\mathrm{blank}\;\mathrm{group}}\right). \end{array}$$

#### Real-Time PCR Analysis

HepG2 cells were plated in 6-well plates and then divided into four groups as 10% saline-treated rat serum group (control group), 10% low dosage FA-medicated serum group, 10% medium dosage FA-medicated serum group, and 10% high dosage FA-medicated serum group. Cells were pre-incubated with the corresponding sera for 24 h. At the terminal of treatment, total RNAs were extracted with Trizol Reagent in accordance with the manufacturer’s instruction. The concentration of RNA was quantified and the quality of RNA was assessed through the ratio of the absorbance at 260 and 280 nm. Subsequently, 2 μg of RNA was reversely transcripted to cDNA using RevertAid First Strand cDNA Synthesis kit, and then the cDNA was amplified using All-in-One^TM^ qPCR Mix Kit. The sequences of the primers are shown in **Table 2**. The reactions were conducted with a total reaction volume of 20 μl containing 2 μl of template diluted (1:10). The program of RT-PCR was set as follow: 95°C for 10 min, 40 cycles of 95°C for 15 s, 58°C for 20 s, and 72°C for 45 s. The relative mRNA expression was analyzed by the 2^-ΔΔ^*^C^*^t^ method.

**Table 2:** The RT-PCR oligonucleotide primers in HepG2 cells.

Gene	Primer	Sequence (5′–3′)	PCR product (bp)
CYP2E1	Forward	CTCGTGGAAATGGAGAAGGA	112
	Reverse	TTGTGCTGGTGGTCTCTGTC	
CYP3A4	Forward	CAAGACCCCTTTGTGGAAAA	184
	Reverse	CGAGGCGACTTTCTTTCATC	
CYP1A2	Forward	GGGCACTTCGACCCTTACAA	63
	Reverse	GCACATGGCACCAATGACG	
β-actin	Forward	GGGCACGAAGGCTCATCATT	295
	Reverse	AGTCGGTTGGAGCGAGCATC	

### Statistical Analysis

Statistical analysis was performed with the SPSS 17.0 (SPSS Inc., Chicago, IL, United States). Data are expressed as mean ± SD. Pharmacokinetic analysis of data was calculated by DAS 2.0 (Mathematical Pharmacology Professional Committee of China, Shanghai, China) and statistically significant difference between groups was assessed by a two-tailed, two sample *t*-test. Other data were analyzed by one-way analysis of variance (ANOVA). A *P*-value less than 0.05 were considered as the statistical significant difference.

## Results

### Effect of FA on CYPs Protein Expressions in Rat Liver

**FIGURE 1:**
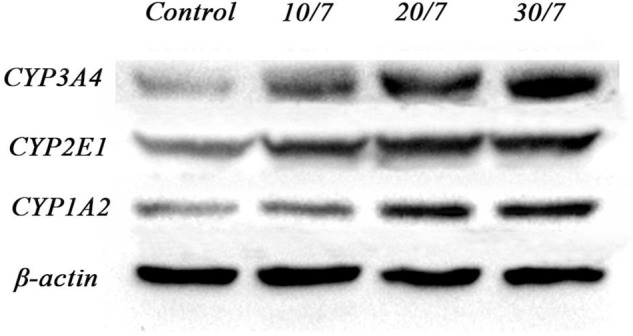
Grayscale plot of protein expression ration of CYP1A2, CYP3A4, and CYP2E1 in rat liver determined by Western Blot. Control, the control group treated with saline; 10/7, low-dose group received 10 g kg^-1^day^-1^ FA extraction; 20/7, medium-dose group received 20 g kg^-1^day^-1^ FA extraction; and 30/7, high-dose group received 30 g kg^-1^day^-1^ FA extraction.

**FIGURE 2:**
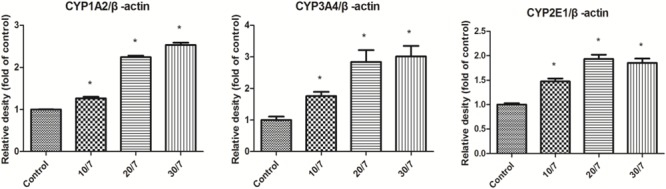
Relative protein expression ration of CYP1A2, CYP3A4, and CYP2E1 in rat liver determined by Western Blot. Control, the control group treated with saline; 10/7, low-dose group received 10 g kg^-1^day^-1^ FA extraction; 20/7, medium-dose group received 20 g kg^-1^day^-1^ FA extraction; and 30/7, high-dose group received 30 g kg^-1^day^-1^ FA extraction. Values are means ± SD and ^∗^*P* < 0.05 compared with control group.

As demonstrated in **Figures 1, 2**, Western blot results of CYP1A2, CYP2E1, and CYP3A4 indicated visible upregulation of protein expression in rat liver compared to saline-treated group. CYP1A2 and CYP3A4 protein expression was significantly induced in all treatment conditions. Similarly, the protein expression of CYP2E1 was significantly up-regulated.

### Effect of FA on CYPs mRNA Expression in Rat Liver

**FIGURE 3:**
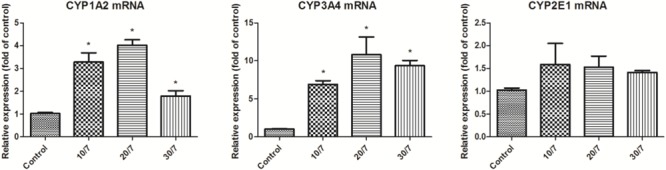
CYP1A2, CYP3A4, and CYP2E1 mRNA expression in rat liver after orally administration of FA. Control, the control group treated with saline; 10/7, low-dose group received 10 g kg^-1^day^-1^ FA extraction; 20/7, medium-dose group received 20 g kg^-1^day^-1^ FA extraction; and 30/7, high-dose group received 30 g kg^-1^day^-1^ FA extraction. Values are means ± SD and ^∗^*P* < 0.05 compared with control group.

The effects of FA on CYP1A2, CYP2E1, and CYP3A4 mRNA expression in rat liver were determined by RT-PCR method. As shown in **Figure 3**, compared with the control group, the mRNA expression of CYP1A2 was significantly induced, especially at the high dosage group; FA seemed to increase CYP3A4 mRNA expression in a dose-dependent manner and statistically significant increase were observed at all treatment conditions, which is consistent with the corresponding CYP protein expression. However, there were not significant changes in CYP2E1 mRNA expression.

### Effect of FA on the CYPs Activities in Rats

**FIGURE 4:**
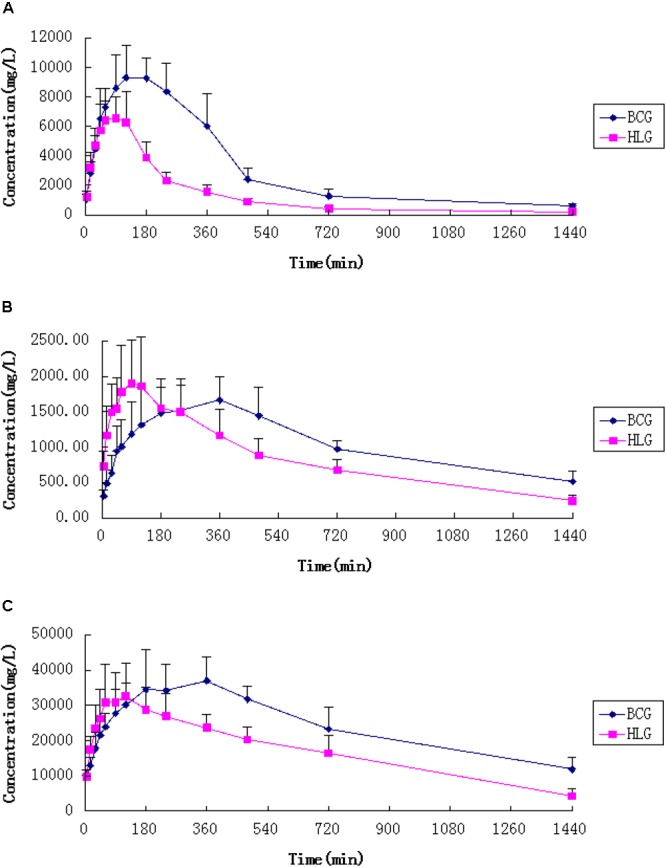
Mean plasma concentration-time curves of caffeine **(A)**, dapsone **(B)**, and chlorzoxazonee **(C)** in untreated and FA extract pre-treated rats (*n* = 6). Blank control group (BCG) received saline for 7 days; HLG (experimental group) received 30 g kg^-1^day^-1^ FA extraction. Error bars represent SD.

The concentrations of caffeine, dapsone, and chlorzoxazone in rat plasma were determined by a sensitive and simple UPLC method. CYP1A2, CYP3A4, and CYP2E1 activities were evaluated by comparing pharmacokinetic parameters of caffeine, dapsone, and chlorzoxazone in control and experimental group. The main pharmacokinetic parameters of caffeine, dapsone, and chlorzoxazone in rats are presented in **Table 3**. Mean plasma concentration–time curves of caffeine, dapsone, and chlorzoxazone are presented in **Figure 4**. As demonstrated in **Table 3**, compared with the control group, the t_1/2_, C_max_, AUC(0-∞), and MRT(0-∞) of caffeine and dapsone in experimental group were decreased significantly (*P* < 0.05); CL of caffeine and dapsone in experimental group were increased significantly (*P* < 0.05). The results indicated that the metabolisms of caffeine and dapsone in experimental group were evidently accelerated and that potential induction of rat hepatic CYP1A2 and CYP3A4 activities was evidence-based in presence of FA. However, no significant differences for the pharmacokinetic parameters of chlorzoxazone (*P* > 0.05) between the control group and the experimental group were observed, which indicated that FA was not able to induce or inhibit rat hepatic CYP2E1 activity *in vivo*.

**Table 3:** Main pharmacokinetic parameters of caffeine, dapsone, and chlorzoxazone in rat plasma.

Probe drug	Pharmacokinetics	Control group	Experimental group
Caffeine	t_1/2_(h)	6.76 ± 3.59	4.06 ± 1.99^∗^
	C_max_ (mg L^-1^)	9.92 ± 1.82	7.06 ± 1.66^∗∗^
	AUC(0-∞) (mg L^-1^ h)	77.50 ± 16.88	31.75 ± 5.35^∗∗^
	MRT(0-∞) (h)	8.05 ± 2.02	5.50 ± 1.59^∗^
	CL (L h^-1^ kg^-1^)	0.13 ± 0.03	0.32 ± 0.06^∗∗^
Dapsone	t_1/2_ (h)	11.58 ± 4.72	8.59 ± 3.52^∗^
	C_max_ (mg L^-1^)	1.74 ± 0.39	2.01 ± 0.69
	AUC(0-∞) (mg L^-1^ h)	33.52 ± 8.10	22.83 ± 3.46^∗∗^
	MRT(0-∞) (h)	17.94 ± 6.60	12.1 ± 4.03
	CL (L h^-1^ kg^-1^)	0.31 ± 0.08	0.45 ± 0.06^∗∗^
Chlorzoxazone	t_1/2_ (h)	1.29 ± 0.95	0.98 ± 0.63
	C_max_ (mg L^-1^)	10.90 ± 2.25	9.56 ± 1.50
	AUC(0-∞) (mg L^-1^ h)	39.74 ± 3.97	22.71 ± 3.91^∗^
	MRT(0-∞) (h)	3.42 ± 0.25	4.53 ± 1.58
	CL (L h^-1^ kg^-1^)	0.51 ± 0.05	0.90 ± 0.16^∗^

*t_*1/2*_, half-life; C_*max*_, the peak or maximum concentration; AUC(0-∞), area under a curve extrapolated to infinity; MRT(0-∞), mean residence time; CL, the total body clearance. Values are presented as mean ± SD (*n* = 6).^∗^*P* < 0.05 when compared with related parameters of control group. ^∗∗^*P* < 0.01 when compared with related parameters of control group.*

### UPLC Analysis of FA-Medicated Serum

**FIGURE 5:**
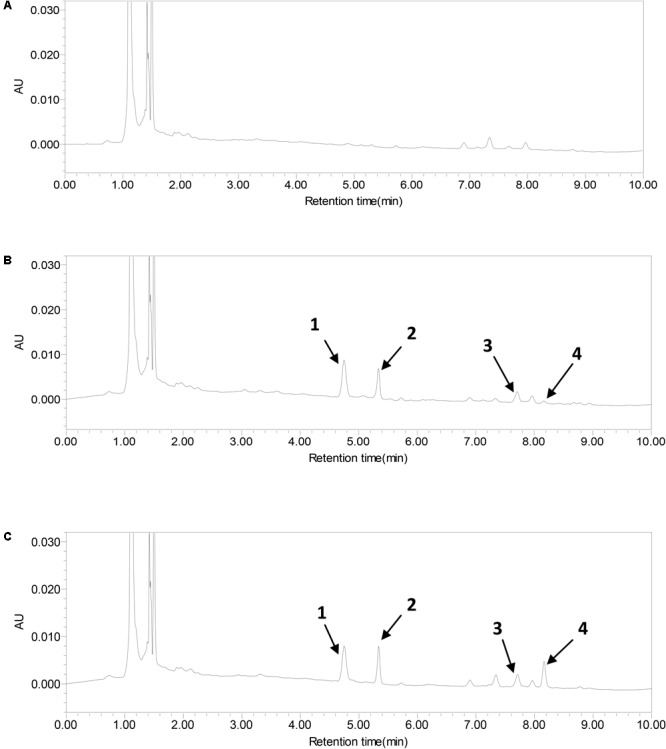
UPLC-PAD chromatograms of sulfamethoxazole (1), meranzin hydrate (2), naringenin (3), and hesperetin (4) in serum. **(A)** Blank serum, **(B)** FA-mediated serum, and **(C)** blank serum added the standard substances.

The UPLC chromatogram implied that there were complex components with several peaks at different retention times in the FA-medicated serum (**Figure 5**), those components including prototype drug component and their metabolites. The retention time of reference standards (meranzin hydrate, naringenin, and hesperetin) was 5.3, 7.8, 8.1 min, respectively. With regard to retention time and UV wavelength of reference substances, it was verified that the FA-medicated serum contained meranzin hydrate, naringenin, and hesperetin.

### Effects of FA-Medicated Serum on Cell Viability

The MTT assay was applied to assess the effect of FA-medicated serum on HepG2 cell viability, which was presented in **Table 4**. Results showed that low dosage, medium dosage, and high dosage FA-medicated serum had no statistical significant influence on cell viability (*P* > 0.05) compared to the control group. Thus, the serum could be used for further study.

**Table 4:** Effects of FA-medicated serums on cell viability of HepG2 cells.

Concentration (g kg^-1^)	FA-medicated serum			Control group
	l0 (low dosage)	20 (medium dosage)	30 (high dosage)	/
OD value Cell viability (%)	0.897 ± 0.166	0.838 ± 0.113	0.944 ± 0.144	0.997 ± 0.053
	89.98	84.07	94.66	100.00
*P*-value	0.488245	0.144198	0.654195	/

*The absorbance value at 570 nm (mean ± SD, *n* = 5).*

### Expression of CYP450 Isoenzymes mRNA Expression in HepG2 Cells Cultured With FA-Mediated Serum

**FIGURE 6:**
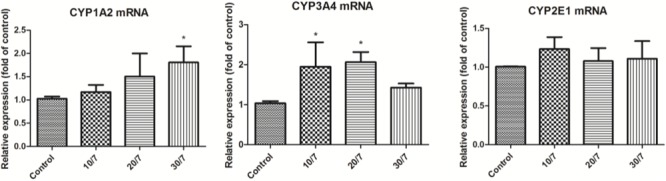
CYP1A2, CYP3A4, and CYP2E1 mRNA expression in HepG2 cells cultured with FA-mediated serum using real-time PCR. Control, 10% saline-treated rat serum group; 10/7, 10% low dosage FA-medicated serum group; 20/7, 10% medium dosage FA-medicated serum group; and 30/7, 10% high dosage FA-medicated serum group. Results are expressed as mean ± SD and ^∗^*P* < 0.05 compared with the control.

The RT-PCR analysis, which was shown in **Figure 6**, was performed to determine the effects of FA-medicated serums on CYP1A2, CYP3A4, and CYP2E1 mRNA expression in HepG2 cells. FA-medicated serum seemed to increase CYP1A2 mRNA expression in a dose-dependent manner; statistically significant increase was observed at the high dosage group. The mRNA expression of CYP3A4 was significantly induced when HepG2 cells were treated with 10% low and medium dosage FA-medicated serum, except 10% high dosage FA-medicated serum. However, there was very small, not significant change in CYP2E1 mRNA expression.

## Discussion

It is estimated that up to 80% of the population in developing countries use traditional herbal medicine for primary health care (Picking et al., 2011). In other word, Herbal medicines play important roles in the primary health care of individuals and communities. But there is a potential risk of unexpected drug–drug interactions when patients administrate herbs and prescribed modern medication simultaneously without informing their physician of their herb use (Ooi et al., 2011). The classical herb implicated with drug–drug interactions is St. John’s wort (Sim and Levine, 2010; Ooi et al., 2011). Pharmacokinetic interaction is one category of drug–drug interaction (Pal and Mitra, 2006), which mainly attributed to the induction or inhibition of specific CYPs. The CYPs superfamily is one of the most important drug-metabolizing enzyme systems and involves in the biotransformation of a large number of exogenous and endogenous compounds (Wang et al., 2015). Depending on how the drugs interact with CYPs, it can be beneficial by enhancing blood levels or detrimental leading to therapeutic failure (Pal and Mitra, 2006). For example, chronic administration of certain herbal products can enhance the production of CYPs, resulting in lowering plasma concentrations due to either higher metabolism and/or higher efflux. Such interactions may lead to lower bioavailability and subtherapeutic plasma drug concentrations (Kumar et al., 2010). In this study, we investigated the effects of the herb FA on hepatic CYP1A2, CYP2E1, and CYP3A4.

In our study, FA significantly induced CYP2E1 protein expression, but did not appear to have significant effect on the corresponding mRNA expression in rat liver. Previous studies have proved that the expression of specific mRNAs and subsequent accumulation of corresponding proteins are not always in accordance (Nakaminami et al., 2014). The research has demonstrated that the CYP3A4 mRNA level correlated significantly to the CYP3A4 protein level, which was not the case for CYP2E1 (Sumida et al., 1999). It has also been demonstrated that CYPs in the liver usually are regulated at the transcriptional level except for CYP2E1. Despite that the mRNAs are the source of protein content, protein expression, namely CYP2E1, can be regulated at various levels including pre-transcription, transcription, pre-translation, translation, and post-translation (Sumida et al., 1999). This may explain our results that CYP2E1 mRNA expression and its corresponding protein expression is not completely consistent. It is reported that the most common mechanism of CYP450 induction is transcriptional activation resulting in the increased protein synthesis of CYP enzymes (Døssing et al., 1983; Han et al., 2014). Thus, For CYP2E1, our result indicates that FA is unlikely to significantly affect CYP2E1 until the clinical researches are conducted. For CYP1A2 and CYP 3A4, the present results showed that FA up-regulated CYP1A2 and CYP3A4 significantly not only from the protein expression but also from the mRNA expression in rat liver. This suggests that FA is potential to induce CYP1A2 and CYP3A4 before completing the clinic trial. Another reason for our conclusions was based on the fact that FA had the potential to induce rat hepatic CYP1A2 and CYP3A4 activities responsible for relevant alterations of probe drug plasma levels, and was not able to induce or inhibit rat hepatic CYP2E1 activity in the experiment. These imply that the potential risk of drug–drug interaction will occur when FA is consumed accompanied with CYP1A2 or CYP3A4 substrates. Conversely, there is low probability of pharmacokinetic interaction between FA and substrates of CYP2E1.

The CYPs mRNA expression in HepG2 cell is based on HepG2 cell model, serum pharmacological method, UPLC method, and MTT assay for reasons as follow: first, HepG2 cells, the human liver cancer cell with different gene expression profiles, are known to constitutively express CYP1A2, CYP2E1, and CYP3A4 (Ooi et al., 2011). Besides, HepG2 cell model is easy to obtain, control, and perform without the inter-individual and inter-species variation and interpretation difficulties, which could accurately evaluate the potential inductive effect for drugs (Cui et al., 2014). Second, Serum pharmacological method is frequently adapted to the study of herb medicines, as it not only eliminates the interferences of the physical and chemical characters of crude drugs on the experimental results, but also provides the experimental conditions similar to the *in vivo* environment (Yin et al., 2014). To replicate the required experimental environment, the 10% (v/v) FA-medicated serum, which is proved statistical non-toxic by MTT assay (**Table 4**) was used for our study. Third, UPLC-PDA-Q-TOF/MS analysis of constituents absorbed into blood of FA extract indicates that flavonoid glycosides, including naringin, hesperidin, and neohesperidin were main components absorbed *in vivo* (Ma et al., 2011). It has been reported that the AUC0-t of naringenin and hesperetin were much higher than those of flavanone glycosides in rat plasma following oral administration of FA extract, which might result from the hydrolysis of flavanone glycosides (naringin, hesperidin, and neohesperidin) (Tong et al., 2012). Meranzin hydrate was considered as one major component of FA and induced similar effect to FA on intestinal motility (Huang et al., 2011). Hence, in our present study, naringenin, hesperetin, and meranzin hydrate were selected to be detected as the bioactive compounds in FA-mediated rat serum (**Figure 1**). Fourth, the result of MTT assay showed that FA-medicated serum at the three different concentrations did not exhibit significant cytotoxicity against HepG2 cells, which exclude the effect of the variable-the activity and number of HepG2 cells on the result of PCR analysis. In our study, FA-medicated serum increased CYP1A2 and CYP3A4 mRNA expression with statistically significant difference compared to the control group, while there was not significant change in CYP2E1 mRNA expression. This result supported the conclusion get in rat above.

A previous study showed that water extract of FA immature increased CYP3A4 protein expression and ethanol extract of FA immature induced CYP3A4 expression via induction of PXR expression (Okada et al., 2017). They speculated that narirutin might be the key constituent increasing PXR expression and CYP3A4 protein expression. It is well known that the efficacy of herbs is significantly correlated with active constituents in herbs. Narirutin is also one of the major flavonoids in FA and thought to be biological active (Huang et al., 2012). It has similar effects to FA to some extent (Huang et al., 2012). Thus, narirutin might be a main compound contributing to the slight induction of FA on CYPs. This needs further experiments to test the hypothesis.

The CYP enzyme induction, one of the common cases of metabolic enzymes related to drug–drug interaction beside the drug metabolism inhibition, causes serious clinical consequences (Cui et al., 2014). For drugs that produce therapeutic effects primarily by the prototype drug, induction may result in a reduced therapeutic efficacy of co-administrated drugs, or even lead to therapy failure (Lin, 2006). For that, the dosage of drugs should be modified within their safe range clinically. For example, rifampin interacts with warfarin resulted from its enzyme inducer activity on CYP2C9, CYP3A4, CYP1A2, and CYP2C19. There was a case report that a female on anticoagulation increased warfarin dose from 52.5 to 210 mg/week with a target INR during the 6-week treatment with rifampin for her infective endocarditis and she was stable on the warfarin dose of 80 mg/week 5 weeks after the end of rifampin therapy (Fahmi et al., 2016). Multiple dose adjustment may occur when FA is combined with CYP1A2 or CYP3A4 substrates with a narrow therapeutic index. Besides, induction may increase the risk of metabolite-induced toxicity by increasing reactive metabolite formation, such as CYP1A induction (Lin, 2006).

## Conclusion

Our study shows that FA may be a potential inducer of CYP1A2, CYP3A4, and may not be able to affect CYP2E1 until the clinic researches are performed. Thus, caution should paid to reduce adverse drug–drug interaction when FA is administrated combined with CYP1A2 or CYP3A4 substrates.

## Data Availability Statements

All the relevant data are contained within the manuscript.

## Author Contributions

XQ contributed the conception and the design of this study. LZ and MC conducted the experiments. LZ and XQ wrote the main manuscript text. All the authors participated in performing the laboratory analyses and interpreting the data. All the authors read and approved the final manuscript.

## Conflict of Interest Statement

The authors declare that the research was conducted in the absence of any commercial or financial relationships that could be construed as a potential conflict of interest.

## References

[B1] Bièche I. Narjoz C. Asselah T. Vacher S. Marcellin P. Lidereau R. (2007). Reverse transcriptase-PCR quantification of mRNA levels from cytochrome (CYP)1, CYP2 and CYP3 families in 22 different human tissues. *Pharmacogenet. Genomics* 17 731–742. 10.1097/FPC.0b013e32810f2e58 17700362

[B2] Cui H. M. Zhang Q. Y. Wang J. L. Chen J. L. Zhang Y. L. Tong X. L. (2014). In vitro studies of berberine metabolism and its effect of enzyme induction on HepG2 cells. *J. Ethnopharmacol.* 158 388–396. 10.1016/j.jep.2014.10.018 25456436

[B3] Døssing M. Pilsgaard H. Rasmussen B. Poulsen H. E. (1983). Time course of phenobarbital and cimetidine mediated changes in hepatic drug metabolism. *Eur. J. Clin. Pharmacol.* 25 215–222. 10.1007/BF00543794 6628504

[B4] Fahmi A. M. Abdelsamad O. Elewa H. (2016). Rifampin-warfarin interaction in a mitral valve replacement patient receiving rifampin for infective endocarditis: a case report. *Springerplus* 5:8. 10.1186/s40064-015-1653-8 26759747 PMC4700031

[B5] Fan R. Huang X. Wang Y. Chen X. Ren P. Ji H. (2012). Ethnopharmacokinetic- and activity-guided isolation of a new antidepressive compound from *Fructus aurantii* found in the traditional Chinese medicine Chaihu-Shugan-San: a new approach and its application. *Evid. Based Complement. Alternat. Med.* 2012:607584. 10.1155/2012/607584 22454671 PMC3291166

[B6] Guo L. Q. Taniguchi M. Chen Q. Y. Baba K. Yamazoe Y. (2001). Inhibitory potential of herbal medicines on human cytochrome P450-mediated oxidation: properties of umbelliferous or citrus crude drugs and their relative prescriptions. *Jpn. J. Pharmacol.* 85 399–408. 10.1254/jjp.85.399 11388644

[B7] Han S. Y. Zhao H. Y. Zhou N. Zhou F. Li P. P. (2014). *Marsdenia tenacissima* extract inhibits gefitinib metabolism *in vitro* by interfering with human hepatic CYP3A4 and CYP2D6 enzymes. *J. Ethnopharmacol.* 151 210–217. 10.1016/j.jep.2013.10.021 24157377

[B8] Hodek P. Trefil P. Stiborová M. (2002). Flavonoids-potent and versatile biologically active compounds interacting with cytochromes P450. *Chem. Biol. Interact.* 139 1–21. 10.1016/S0009-2797(01)00285-X 11803026

[B9] Huang W. Huang X. Xing Z. Qiu X. Wang Y. Fan R. (2011). Meranzin hydrate induces similar effect to *Fructus aurantii* on intestinal motility through activation of H1 histamine receptors. *J. Gastrointest. Surg.* 15 87–96. 10.1007/s11605-010-1374-9 21061180

[B10] Huang W. Xiong Z. H. Huang X. Chen X. Liu W. P. Wang Y. (2012). Simultaneous UPLC analysis of three major flavonoids in granule decoctions of *Fructus aurantii*-type formulae. *Pharmazie* 67 586–589. 22888512

[B11] Huang Y. T. Wang G. F. Chen C. F. Chen C. C. Hong C. Y. Yang C. (1995). *Fructus aurantii* reduced portal pressure in portal hypertensive rats. *Life Sci.* 57 2011–2020. 10.1016/0024-3205(95)02195-O 7475952

[B12] Kang M. Kim J. H. Cho C. Chung H. S. Kang C. W. Kim Y. (2007). Anti-ischemic effect of *Aurantii Fructus* on contractile dysfunction of ischemic and reperfused rat heart. *J. Ethnopharmacol.* 111 584–591. 10.1016/j.jep.2007.01.007 17291701

[B13] Kim J. H. Chung H. S. Kang M. Kim Y. Kim Y. S. Bae H. (2011). Anti-diabetic effect of standardized herbal formula PM021 consisting of mori folium and *Aurantii fructus* on type II diabetic Otsuka Long-Evans Tokushima Fatty (OLETF) rats. *Diabetes Res. Clin. Pract.* 93 198–204. 10.1016/j.diabres.2011.03.037 21524812

[B14] Kumar Y. S. Adukondalu D. Sathish D. Vishnu Y. V. Ramesh G. Latha A. B. (2010). P-Glycoprotein- and cytochrome P-450-mediated herbal drug interactions. *Drug Metabol. Drug Interact.* 25 3–16. 10.1515/DMDI.2010.006 21417789

[B15] Li X. Xiao H. Liang X. Shi D. Liu J. (2004). LC–MS/MS determination of naringin, hesperidin and neohesperidin in rat serum after orally administrating the decoction of *Bupleurum falcatum* L. and *Fractus aurantii*. *J. Pharm. Biomed. Anal.* 34 159–166. 10.1016/j.japna.2003.08.002 14738930

[B16] Lin J. H. (2006). CYP induction-mediated drug interactions: *in vitro* assessment and clinical implications. *Pharm. Res.* 23 1089–1116. 10.1007/s11095-006-0277-7 16718615

[B17] Liu L. Cheng Y. Zhang H. (2004). Phytochemical analysis of anti-atherogenic constituents of Xue-Fu-Zhu-Yu-Tang using HPLC-DAD-ESI-MS. *Chem. Pharm. Bull.* 52 1295–1301. 10.1248/cpb.52.1295 15516749

[B18] Liu Z. Zhang J. Gao W. Liu C. (2013). Antinociceptive activity and chemical composition of Wei-Chang-An-Wan extracts. *Pharm. Biol.* 51 790–797. 10.3109/13880209.2013.766893 23675838

[B19] Ma C. Gao W. Gao Y. Man S. Huang L. Liu C. (2011). Identification of chemical constituents in extracts and rat plasma from *Fructus aurantii* by UPLC-PDA-Q-TOF/MS. *Phytochem. Anal.* 22 112–118. 10.1002/pca.1252 21259373

[B20] Nakaminami K. Matsui A. Nakagami H. Minami A. Nomura Y. Tanaka M. (2014). Analysis of differential expression patterns of mRNA and protein during cold-acclimation and de-acclimation in *Arabidopsis*. *Mol. Cell. Proteomics* 13 3602–3611. 10.1074/mcp.M114.039081 25277243 PMC4256508

[B21] Okada N. Murakami A. Urushizaki S. Matsuda M. Kawazoe K. Ishizawa K. (2017). Extracts of immature orange (*Aurantii fructus immaturus*) and Citrus Unshiu Peel (*Citri unshiu pericarpium*) induce P-glycoprotein and cytochrome P450 3A4 expression via upregulation of pregnane X receptor. *Front. Pharmacol.* 8:84. 10.3389/fphar.2017.00084 28270768 PMC5318391

[B22] Ooi J. P. Kuroyanagi M. Sulaiman S. F. Muhammad T. S. Tan M. L. (2011). Andrographolide and 14-deoxy-11, 12-didehydroandrographolide inhibit cytochrome P450s in HepG2 hepatoma cells. *Life Sci.* 88 447–454. 10.1016/j.lfs.2010.12.019 21219911

[B23] Pal D. Mitra A. K. (2006). MDR- and CYP3A4-mediated drug-drug interactions. *J. Neuroimmune Pharmacol.* 1 323–339. 10.1007/s11481-006-9034-2 18040809

[B24] Picking D. Younger N. Mitchell S. Delgoda R. (2011). The prevalence of herbal medicine home use and concomitant use with pharmaceutical medicines in Jamaica. *J. Ethnopharmacol.* 137 305–311. 10.1016/j.jep.2011.05.025 21645607

[B25] Sim S. N. Levine M. A. (2010). An evaluation of pharmacist and health food store retailer’s knowledge regarding potential drug interactions associated with St. John’s wort. *Can. J. Clin. Pharmacol.* 17 e57–e63. 20147772

[B26] Sumida A. Kinoshita K. Fukuda T. Matsuda H. Yamamoto I. Inaba T. (1999). Relationship between mRNA levels quantified by reverse transcription-competitive PCR and metabolic activity of CYP3A4 and CYP2E1 in human liver. *Biochem. Biophys. Res. Commun.* 262 499–503. 10.1006/bbrc.1999.1233 10462503

[B27] Tong L. Zhou D. Gao J. Zhu Y. Sun H. Bi K. (2012). Simultaneous determination of naringin, hesperidin, neohesperidin, naringenin and hesperetin of *Fractus aurantii* extract in rat plasma by liquid chromatography tandem mass spectrometry. *J. Pharm. Biomed. Anal.* 58 58–64. 10.1016/j.jpba.2011.05.001 22018980

[B28] Tydén E. Tjälve H. Larsson P. (2014). Gene and protein expression and cellular localisation of cytochrome P450 enzymes of the 1A, 2A, 2C, 2D and 2E subfamilies in equine intestine and liver. *Acta Vet. Scand.* 56:69. 10.1186/s13028-014-0069-8 25288196 PMC4192735

[B29] Wang Y. Wu S. Chen Z. Zhang H. Zhao W. (2015). Inhibitory effects of cytochrome P450 enzymes CYP1A2, CYP2A6, CYP2E1 and CYP3A4 by extracts and alkaloids of *Gelsemium elegans* roots. *J. Ethnopharmacol.* 166 66–73. 10.1016/j.jep.2015.03.002 25764964

[B30] Wilkinson G. R. (2005). Drug metabolism and variability among patients in drug response. *N. Engl. J. Med.* 352 2211–2221. 10.1056/NEJMra032424 15917386

[B31] Xiong X. Peng W. Chen L. Liu H. Huang W. Yang B. (2015). Traditional Chinese medicine Zhiqiao–Houpu herb-pair induce bidirectional effects on gastric motility in rats. *J. Ethnopharmacol.* 175 444–450. 10.1016/j.jep.2015.10.001 26456365

[B32] Yin J. Luo Y. Deng H. Qin S. Tang W. Zeng L. (2014). Hugan Qingzhi medication ameliorates hepatic steatosis by activating AMPK and PPARα pathways in L02 cells and HepG2 cells. *J. Ethnopharmacol.* 154 229–239. 10.1016/j.jep.2014.04.011 24735863

[B33] Yoshida N. Takagi A. Kitazawa H. Kawakami J. Adachi I. (2005). Inhibition of P-glycoprotein-mediated transport by extracts of and monoterpenoids contained in zanthoxyli fructus. *Toxicol. Appl. Pharmacol.* 209 167–173. 10.1016/j.taap.2005.04.001 15890377

[B34] Zhang Y. J. Huang W. Huang X. Wang Y. Wang Z. Wang C. (2012). *Fructus aurantii* induced antidepressant effect via its monoaminergic mechanism and prokinetic action in rat. *Phytomedicine* 19 1101–1107. 10.1016/j.phymed.2012.05.015 22770641

[B35] Zhou D. Y. Chen D. L. Xu Q. Xue X. Y. Zhang F. F. Liang X. M. (2007). Characterization of polymethoxylated flavones in *Fructus aurantii* by liquid chromatography with atmospheric pressure chemical ionization combined with tandem mass spectrometry. *J. Pharm. Biomed. Anal.* 43 1692–1699. 10.1016/j.jpba.2006.12.032 17291708

